# Comparative Transcriptomic Analysis Reveals Salt Stress Adaptation Mechanisms in Cultivated Rice Varieties (*Oryza sativa*)

**DOI:** 10.3390/cimb48030321

**Published:** 2026-03-18

**Authors:** Zihao Yuan, Ziqi Liu, Shengyu Mo, Feng Wang, Wuge Liu, Dilin Liu, Wu Yang, Yilong Liao, Leiqing Chen, Le Kong, Hui Wang, Tao Guo, Xing Huo

**Affiliations:** 1 College of Agriculture, South China Agricultural University, Guangzhou 510640, China; yuanzihao.scau.edu.cn@stu.scau.edu.cn (Z.Y.); moshengyu2000@163.com (S.M.); wanghui@scau.edu.cn (H.W.); 2 Rice Research Institute, Guangdong Academy of Agricultural Sciences/South China High-Quality Rice Breeding Laboratory (Jointly Established by Ministry of Agriculture and Rural Affairs and Provincial Government)/Guangdong Key Laboratory of Rice Science and Technology/Guangdong Rice Engineering Laboratory, Guangzhou 510640, China; liuziqi12@stu.gdou.edu.cn (Z.L.); wangfeng@gdaas.cn (F.W.); liuwuge@gdaas.cn (W.L.); dilin_liu@163.com (D.L.); yangwu@gdaas.cn (W.Y.); liaoyilong8888@163.com (Y.L.); chenleiq0816@163.com (L.C.); kongldk@163.com (L.K.); 3 College of Coastal Agriculture Science, Guangdong Ocean University, Zhanjiang 524088, China

**Keywords:** salt stress, *Oryza sativa* (rice), germination, transcriptomics, RNA sequence

## Abstract

Salt stress is an injurious concern of global climate change that negatively impacts the growth and yield of rice plants. Identifying salt tolerance genes is essential to understanding the molecular mechanism regulating salt tolerance in rice. In this study, we treated two rice varieties, Xiangxiuzhan (XXZ) and Changxiang (CXG), with 100 mM NaCl to examine the effect on the germination and growth stages. Transcriptome analysis was investigated for changes in gene expression between the two varieties. During the germination stage, the CXG variety had higher germination potential than the XXZ variety, whereas in the growth stage, the XXZ variety showed higher survival efficiency than the CXG variety. Transcriptome analysis showed that the XXZ variety had more DEGs in grains, while CXG displayed greater DEGs in leaves and roots. Gene Ontology (GO) and KEGG pathway showed that beta-alanine metabolism, cutin biosynthesis, and plant hormone signal transduction were over-represented, whereas heatmap analysis showed cellular and environmental signal transduction. This study focuses on the molecular pathways of the salt stress tolerance mechanism of Xiangxiuzhan and Changxiang varieties.

## 1. Introduction

In coastal agricultural regions, salinity is the major threat to crop production [1,2] and impacts cultivable fertile land, which may affect climate change [3,4]. Plants experience both osmotic and ionic stress due to high salinity. Osmotic stress occurs instantly, though the effect of ionic stress develops over time as ions develop in the plants’ environments [5,6]. Osmotic pressure at the roots causes water loss and decreased turgor, which stop cells from expanding and slow plant growth. Cells may become harmed by too many ions, especially Na^+^, which can impair metabolism and photosynthesis and cause oxidative stress. Osmotic stress typically triggers growth and stomatal closure throughout the entire plant. Long-term stress can lead to an initial loss of biomass due to a drop in carbon levels, followed by early aging and cell death due to ion toxicity. Recent research suggests that plants may undergo accelerated senescence under moderate stress to redistribute nutrients to reproductive structures, thereby ensuring the survival of future generations [7]. However, this strategy often results in reduced yields for annual crops. Abiotic stresses such as low temperatures, drought, and salinity pose significant threats to plant growth and productivity. Improper irrigation using brackish water exacerbates secondary soil salinity, making the problem even more severe [8,9].

The FAO land and plant nutrition management service says that salinity affects more than 6% of the world’s land area [10]. Of the 230 million hectares of irrigated land in the world, about 45 million hectares are affected by salinity. Another 32 million hectares of dryland agriculture also experience different levels of salt-related stress [10]. High salt levels in the soil cause osmotic stress, which makes it harder for plants to take up water and slows their growth [11,12]. This causes major problems with cells and metabolism, such as less cell growth, less cell wall synthesis, less protein synthesis, less stomatal conductance, and less photosynthetic activity [13,14,15]. As sodium (Na^+^) and chloride (Cl^−^) ions build up over time, they cause ionic stress by throwing off the balance of Na^+^/K^+^ and Na^+^/Ca^2+^ ratios [16]. This imbalance causes the body to make more reactive oxygen species (ROS), which makes cellular homeostasis even worse and causes oxidative stress [6]. Salinity makes it harder for plants to absorb nutrients and maintain balance in their roots, hindering growth and productivity [8].

Rice (*Oryza sativa* L.) is a major food crop and a model organism for monocot research because its genome is relatively small. It is also very sensitive to environmental stress [17]. Salinity stress is a big problem for rice production around the world, especially in coastal areas and other places where salinity is high. Climate change is making these areas more vulnerable to salt intrusion [18]. Studies have indicated that salinity lowers the amount of dry matter rice produces, its ability to photosynthesise, its fertility, and its grain yield [19]. Furthermore, salt affects the way rice breaks down sugar, which hurts its overall growth and development [20]. Developing and choosing rice varieties that can handle salt is still one of the most important ways to deal with salinity stress and increase rice production. There is a strong link between how well rice can handle salt and how much grain it produces. Because of this, it is very important to determine and describe the genes that make plants tolerant to salt in order to improve stress resistance and breeding [21]. Genomic tools and technologies, like the release of the rice draft genome, transcriptomics, and functional genomics, have helped us learn a lot about how plants tolerate salt [22].

Transcriptome analysis of *O. sativa* japonica Nipponbare has found thousands of genes that respond to salt stress, showing how molecules react [23]. Research on Dongxiang wild rice (*O. rufipogon*) and Brazilian rice cultivars has found that salinity stress affects different molecular pathways, such as differentially expressed transcripts and Gene Ontology analyses [24]. The halophytic species *Oryza coarctata* helps us understand how plants survive in salty environments due to their unique genetic adaptations [25]. The goal of this study is to use deep transcriptome sequencing to identify genes that are expressed differently and learn more about the basic molecular processes that control rice’s ability to handle stress. By exploring these mechanisms, we aim to develop rice varieties that can withstand salt and promote environmentally friendly farming practices. Extensive molecular research is required to understand the ability to tolerate salinity of *O. sativa* species.

## 2. Materials and Methods

### 2.1. Plant Materials and Sample Collection

This experiment was conducted on two rice (*Oryza sativa*) varieties. Xiangxiuzhan (which indicates the XXZ variety) and Changxiang (which indicates the CXG variety) were collected from the Rice Research Institute of Guangdong Academy of Agricultural Sciences, Guangzhou, Guangdong Province, China. Then, a germination and salt treatment experiment was conducted at the Rice Research Institute of Guangdong Academy of Agricultural Sciences, Guangdong Province, China, and metabolites were analyzed by Sangon Biotech (Shanghai, China) Co., Ltd.

### 2.2. The Percentage of Seeds That Germinate After Being Treated with Salt

The rice germination experiment was conducted on two varieties (XXZ and CXG) in accordance with Khan, Muhammad Hafeez Ullah et al. 2014 [26], employing a 100 mM NaCl (pH 5.8) solution as the salt treatment, with control seeds only applied with distilled water, based on previous testing. Each Petri dish contained fifty viable seeds immersed in 30 mL of distilled water, and three biological replicates were maintained for each variety and control under each treatment condition. The emergence of a radicle measuring at least 2 mm was the criterion for germination. We determined the relative germination rate by dividing the germination rate in the salt treatment group after seven days by the germination rate in the control group, then multiplying the result by 100.

### 2.3. The Rate of Seed Survival Under Salt Treatment Was Assessed

The survival rate was evaluated according to the following method described by Sun et al. [27]. Eight seven-day-old seedlings were selected from treatment with five replications and placed into 90 mm Petri dishes that had three layers of tissue paper. Then, only 1× Kimura B nutrient solution was used to grow all plants until the one-leaf, one-heart stage. Once the first leaf began to bend and the second leaf emerged, a total of 140 mL of 100 mM NaCl solution (pH 5.8) was applied daily for seven consecutive days with 1× Kimura B nutrient solution for the treatment plant. Only 1× Kimura B nutrient solution applied to plants served as control. The plants were maintained at 28 °C with 75% relative humidity under a 12 h light and 12 h dark photoperiod. After seven days of treatment, calculate the overall survival rate.

### 2.4. RNA Extraction, Library Construction, and Sequencing

For RNA extraction, samples of grain, leaf, and root from both control and treatment groups of the XXZ and CXG varieties were harvested from multiple individual plants after a three-day treatment with 100 mM NaCl, under controlled growth conditions (Table 1). RNA was extracted using the RNAprep Pure Plant Kit (Tiangen Inc., Beijing, China). Subsequently, mRNAs were isolated employing Dynabeads™ Oligo (dT) 25 (Life Technologies, Carlsbad, CA, USA), while rRNA was removed using the Ribo-Zero™ Magnetic Kit (Illumina, San Diego, CA, USA). Random hexamers were employed to fragment the retrieved mRNAs and reverse-transcribe them into complementary DNAs (cDNAs). These cDNAs were subsequently isolated and ligated with adaptors to facilitate paired-end sequencing. The cDNA library was sequenced using the Illumina HiSeq™ 4000 platform by Gene Denovo Biotechnology Co. (Guangzhou, China). The NCBI Sequence Read Archive (SRA) has deposited the sequencing data under the accession number PRJNA830977.

### 2.5. Unigene De Novo Assembly and Annotation

The raw sequencing data was purified by removing low-quality reads and adaptor sequences. Trinity software (v2.15.1) subsequently organized the clean readings into non-redundant unigenes. Then, we employed multiple databases, including the NCBI nucleotide sequence database (Nt), the non-redundant protein database, the Swiss-Prot database, the Clusters of Orthologous Groups (COG) of proteins, Gene Ontology (GO), and the Kyoto Encyclopedia of Genes and Genomes (KEGG) annotation database, to functionally annotate the assembled unigenes.

### 2.6. Identification of Differentially Expressed Genes (DEGs)

The DESeq (v1.51.6) R package was used for differential gene expression analysis, with an FDR threshold of less than 0.05 and |Log2Fold-Change| greater than 4 to find differentially expressed genes (DEGs). Then, we subjected the identified DEGs to Gene Ontology (GO) enrichment analysis using agriGO 2.0, followed by Kyoto Encyclopedia of Genes and Genomes (KEGG) pathway analysis using KOBAS 3.0.

### 2.7. Differentially Expressed Genes (DEGs) Analysis

The Short Time-series Expression Miner (STEM) program (v1.3.11) was used to analyze differentially expressed genes (DEGs) in grains, leaves, and roots after salt treatment to investigate the temporal expression patterns of conditions (Table 1). KEGG pathway analysis further examined the potential functionalities and enriched pathways of the DEGs identified through STEM analysis.

### 2.8. Heatmap Analysis

The gene expression levels were quantified by using FPKM (fractions per kilobase of transcript per million mapped reads). Then, we created a clustered heatmap after normalizing the data using the zero-to-one scaling method and then visualized it using TBtools (v1.098) software [28] (Chen et al., 2020).

### 2.9. Statistical Analysis

All data underwent analysis of variance based on a completely randomized design model utilizing SPSS 26 software (SPSS, Inc., Chicago, IL, USA). Duncan’s Multiple Range Test (DMRT) was applied to determine significant differences among groups at *p* ≤ 0.05 following one-way ANOVA using SPSS Statistics 26 software.

## 3. Results

### 3.1. Effect of Salt Stress on Growth and Germination Stage

**Figure 1:**
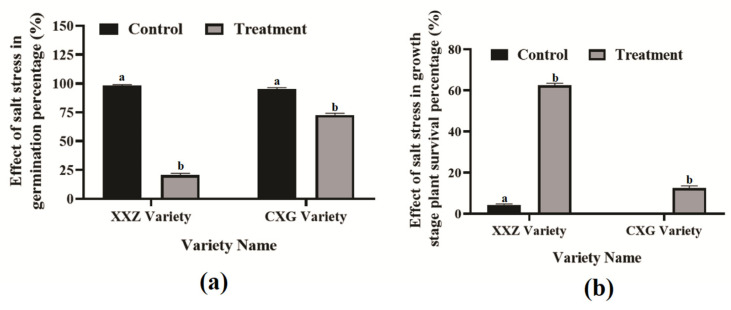
The effect of salt stress on the seed germination and growth stages in XXZ and CXG varieties: (**a**) indicates germination percentage after seven days of NaCl treatment; (**b**) indicates plant survival percentage in growth stage after seven days of NaCl treatment. Different letters indicate significant differences between the mean  ±  SD of replications at a *p*  <  0.05 significance level.

When salt treatment was applied during the germination stage, the XXZ variety showed a germination rate of 20.66%, whereas the CXG variety exhibited a significantly higher germination rate of 72.66% (Figure 1a). In the control variety, the germination rates were 98.00% for XXZ and 95.33% for CXG (Figure 1a). However, when salt treatment was applied at the growth stage, the XXZ variety showed a survival rate of 61.50%, while the CXG variety exhibited a much lower survival rate of 12.50%. Under control variety, the survival rates were 94.17% for XXZ and no survival for CXG (Figure 1b). These results indicate that the salt stress tolerance of the XXZ and CXG varieties shows opposite responses at the germination and growth stages (Figure 1a,b).

### 3.2. Transcriptome Sequencing Data Analysis

**Figure 2:**
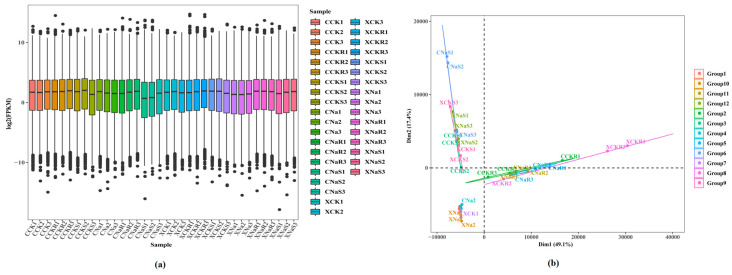
Principal component analysis (PCA) and gene expression level distribution in the transcriptome. (**a**) PCA among the control, XXZ, and the CXG variety; (**b**) gene expression in grains, leaves, and roots under salt stress conditions.

Each sample’s transcriptome (RNA sequence) analysis yielded clean data, with all samples reaching 279.23 GB. Sequence alignment was performed with the reference genome, with alignment efficiency ranging from 85.05% to 95.61%. The base percentage of Q30 was 99.99%. The proportion of bases with a clean data quality value greater than or equal to 30 was above 92%. Under salt stress conditions, there was a significant difference between the two rice varieties (Figure 2). The PCA graph in Figure 2a shows that the samples in each variety were well separated, and there were significant outliers in the internal samples in each period. The use of transcriptome data to detect gene expressions in samples has high sensitivity. The box plot of the gene expression levels in each sample shows the dispersion of the gene expression level distribution in individual samples and allows for comparing the overall gene expression levels in different samples (Figure 2b).

### 3.3. Differentially Expressed Genes (DEGs)

**Figure 3:**
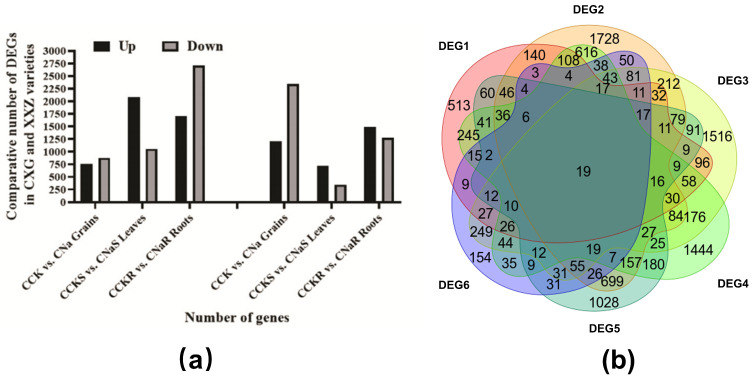
Differentially expressed genes (DEGs) in grains, leaves, and roots of the CXG and XXZ varieties under salt stress. (**a**) Number of up-regulated and down-regulated DEGs under salt stress. (**b**) Venn diagram analysis of the DEGs in CXG grains, CXG roots, CXG leaves, XXZ grains, XXZ roots, and XXZ leaves.

To investigate the expression of differentially expressed genes (DEGs) in response to salinity, cDNA libraries were constructed from samples of grains, leaves, and roots, alongside control varieties CXG and XXZ, for high-throughput RNA sequencing. In the CXG and XXZ varieties, we compared DEGs between the control and treatment groups for grains, leaves, and roots, with three replications. A comparison of the grains from the control and treatment groups of the CXG variety revealed a total of 1632 DEGs, comprising 753 that were up-regulated and 879 that were down-regulated (Figure 3a,b). However, when comparing the leaves of the CXG variety between the control and treatment groups, we identified a total of 3144 DEGs, with 2088 being up-regulated and 1056 down-regulated (Figure 3a,b). Furthermore, a comparison of the roots from the control and treatment groups of the CXG variety resulted in the identification of 4421 DEGs, consisting of 1709 that were up-regulated and 2712 that were down-regulated (Figure 3a,b).

In the XXZ variety, comparing the control and treatment groups in the grains revealed a total of 3558 differentially expressed genes (DEGs). This included 1210 genes that were up-regulated and 2348 genes that were down-regulated (Figure 3a,b). In the leaves, the comparison between the control and treatment groups identified 1066 DEGs, including 721 that were up-regulated and 345 that were down-regulated (Figure 3a,b). Finally, in the roots, the comparison of the control and treatment groups yielded a total of 2774 DEGs, with 1497 up-regulated and 1277 down-regulated (Figure 3a,b).

### 3.4. Gene Ontology (GO)

The differentially expressed genes are salt-responsive genes (SRGs). The SRGs were annotated utilizing the GO (Gene Ontology) database and categorized into three classifications, biological processes, molecular functions, and cellular components (Figure S1), pertinent to rice growth and development. The percentage of DEGs assigned to molecular functions across various control and treatment groups was comparatively low; a greater number of genes were designated to binding and catalytic activities.

### 3.5. KEGG Analysis

**Figure 4:**
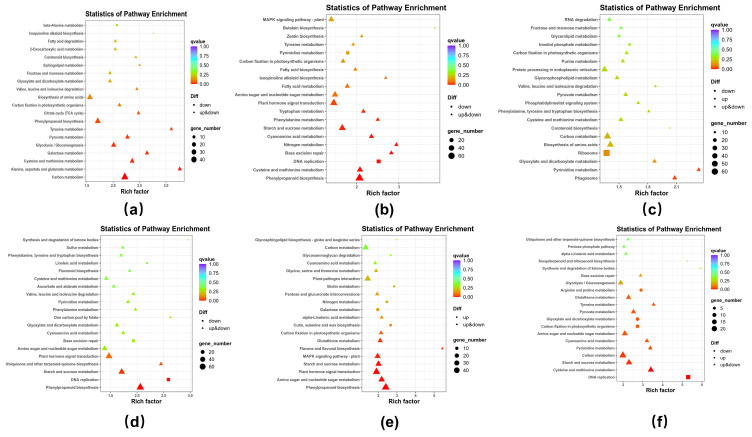
Kyoto Encyclopedia of Genes and Genomes (KEGG) analysis of grains, leaves, and roots of CXG and XXZ varieties under salt stress: (**a**) indicates XCK vs. XNa group in CXG variety, (**b**) indicates CCK vs. CNa group in CXG, (**c**) indicates CCKS and CCKNa group in CXG, (**d**) indicates XCK vs. XNa group in XXZ variety, (**e**) indicates XCKR vs. XNaR group in XXZ, (**f**) indicates XCKS vs. XNaS group in XXZ variety.

In case of the CXG variety, the CCK vs. CNa group showed significant enrichment in genes related to the beta-alanine metabolism pathway, with the majority of 100 DEGs identified in this group (Figure 4a). However, the CCK vs. CNa group did not show significant enrichment in other pathways among the 120 DEGs (Figure 4b). In contrast, the comparison between CCKS and CCKNa revealed significant enrichment in genes involved in the degradation of valine, leucine, and isoleucine, with a total of 210 DEGs significantly enriched in these pathways (Figure 4c).

For the XXZ variety, the XCK vs. XNa group did not show significant enrichment in any pathway, despite the identification of 120 DEGs (Figure 4d). The XCKR vs. XNaR group, however, demonstrated significant enrichment in genes associated with cutin, suberine, and wax biosynthesis, as well as galactose metabolism and biotin pathways, with 100 DEGs identified in total (Figure 4e). In the XCKS vs. XNaS group, DEGs related to plant growth regulator (PGR) responses were primarily enriched in pathways such as carbon fixation in photosynthetic organisms, glyoxylate and dicarboxylate metabolism, arginine and proline metabolism, the pentose phosphate pathway, and ubiquinone and other terpenoid–quinone biosynthesis, involving a total of 50 DEGs (Figure 4f).

### 3.6. Heatmap Analysis

**Figure 5:**
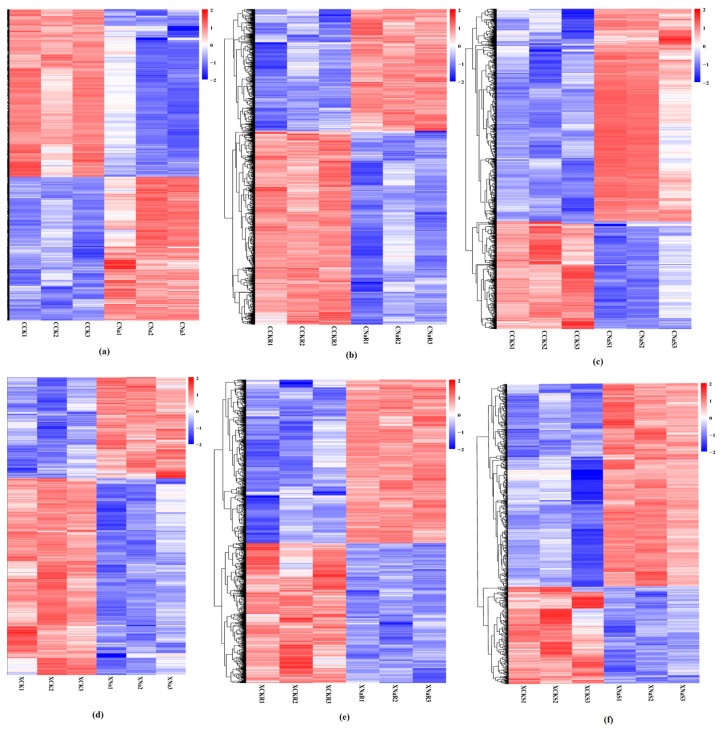
Heatmap of the relative expression of the DEGs involved in grains, leaves, and roots of CXG and XXZ variety under salt stress: (**a**) indicates CCK vs. CNa group in CXG variety, (**b**) indicates CCKR vs. CNa group in CXG, (**c**) indicates CCKS and CNaS group in CXG, (**d**) indicates XCK vs. XNa group in XXZ variety, (**e**) indicates XCKR vs. XNaR group in XXZ, (**f**) indicates XCKS vs. XNaS group in XXZ variety. The clustered heatmap was portrayed after normalized with zero-to-one scale method using the TBtools software.

Regarding plant hormone signal transduction, 562 were genes related to CCK vs. CNa, 874 were CCKR vs. CNaR, 896 were CCKS vs. CNaS, 822 were XCK vs. XNa, 600 were XCKR vs. XNaR, and 337 were XCKS vs. XNaS genes that were related to these signal transduction pathways (Figure 5). Among the 562 genes, 16 were related to cellular signal transduction, 30 were linked to environmental information processing signal transduction, 75 were related to genetic information processing signal transduction, 426 were related to metabolism signal transduction, and 15 were related to organismal system signal transduction (Figure 5a). Among the 874 genes, 35 were related to cellular signal transduction, 50 were linked to environmental information processing signal transduction, 169 were related to genetic information processing signal transduction, 594 were related to metabolism signal transduction, and 26 were related to organismal system signal transduction (Figure 5b). Among the 896 genes, 56 were related to cellular signal transduction, 46 were linked to environmental information processing signal transduction, 205 were related to genetic information processing signal transduction, 560 were related to metabolism signal transduction, and 29 were related to organismal system signal transduction (Figure 5c). Among the 822 genes, 42 were related to cellular signal transduction, 59 were linked to environmental information processing signal transduction, 147 were related to genetic information processing signal transduction, 537 were related to metabolism signal transduction, and 37 were related to organismal system signal transduction (Figure 5d). Among the 600 genes, 27 were related to cellular signal transduction, 42 were linked to environmental information processing signal transduction, 33 were related to genetic information processing signal transduction, 468 were related to metabolism signal transduction, and 30 were related to organismal system signal transduction (Figure 5e). Among the 337 genes, eight were related to cellular signal transduction, 16 were linked to environmental information processing signal transduction, 53 were related to genetic information processing signal transduction, 254 were related to metabolism signal transduction, and six were related to organismal system signal transduction (Figure 5f).

## 4. Discussion

The growing global population and escalating impacts of climate change pose significant challenges to the sustainability of cultivated crops, necessitating innovative approaches in agricultural practices to ensure food security. Due to global warming, salt concentration is rapidly increasing in the land of coastal regions [1]. High salinity is a major abiotic stress that reduces the growth, development, and production of crops. Rice, being a sessile crop plant, has evolved diverse physiological, biochemical, and molecular mechanisms to cope with sudden salt stress. In our present study, when seeds were treated with salt, the CXG variety showed a higher germination rate than that of the XXZ variety. However, in the growth stage, the survival rate of the XXZ variety was higher than the CXG variety. The resistance to salinity depends on several traits, which have been extensively studied in recent years [29].

Although numerous studies have demonstrated salt resistance in genetically engineered rice under laboratory conditions, comprehensive gene expression studies investigating the full spectrum of salt stress responses in rice remain limited. This gap may be due to an incomplete understanding of the full genome range of salt stress responses in rice. The findings offer new insights into the role of response in triggering salt tolerance mechanisms that contribute to the adaptation and genetic improvement of salt tolerance. Research has shown that salt-tolerant varieties distribute sodium unevenly across the plant: higher concentrations are found in the roots compared to the leaves, and more sodium is present in the first leaf than in the second or third [29,30,31,32,33]. Genome-wide identification and functional prediction studies have been conducted across various crop species, including rice (*Oryza sativa*), maize (*Zea mays*), and poplar (*Populus* spp.) [34,35,36]. Using transcriptomics, we assessed the molecular response to salt stress in grains, leaves, and roots after 3 days of exposure, a period when both varieties began activating their distinct stress response programs. The salinity stress tolerance is a complex quantatitative trait governed by multiple genes [37,38]. RNA-seq has emerged as a powerful tool for transcriptomic analysis, allowing the identification of genes and pathways central to the salinity tolerance mechanism in plants. In this experiment, RNA libraries were sequenced to characterize the transcriptomic response to salinity stress in rice varieties Xiangxiuzhan and Changxiang which are widely distributed and agronomically valuable. The analysis of differentially expressed genes revealed a more extensive response in the CXG variety, with more genes being differentially expressed compared to the XXZ variety. In the CXG variety, grain, leaves, and roots revealed a total of 1632, 3144, and 4421 DEGs whereas in the XXZ variety, grains, leaves, and roots showed 3558, 1066, and 2774 DEGs. High-throughput RNA-seq technology has not only enhanced genome annotation efforts in rice but also provided insight into gene expression dynamics under stress conditions.

Fundamental analysis using Gene Ontology (GO) classification revealed that differentially expressed genes (DEGs) were enriched in pathways associated with hormone signaling, calcium signaling, transduction factors, ion transport, nitrogen metabolism, and secondary metabolism. The first group of DEGs linked to salinity stress responses was associated with plant hormone and calcium signaling pathways. Salinity stress imposes both osmotic and ionic challenges on plants. The direct toxicity of Na^+^ ions including the disruption of K^+^/Na^+^ homeostasis, interference with enzymatic functions, and induction of oxidative stress plays a critical role in inhibiting plant growth and development [6]. These ion-specific effects are superimposed upon the initial osmotic stress caused by reduced water potential in the root environment. Consequently, salinity triggers a complex, biphasic growth response in plants, characterized by an initial period of osmotic stress-induced growth inhibition followed by longer-term ionic toxicity effects that may determine survival and adaptive capacity. Transcription factors are crucial for changing how plants respond to stress. Salinity stress creates osmotic and ionic imbalances, which have a big effect on how ions are balanced in the roots. Stress-inducible genes that help move ions, like the membrane pore gene, were turned up under salty conditions. After stress, the two genotypes had different ways of controlling nitrogen metabolism pathways. Genes linked to nitrogen pathways exhibited stability during initial stress phases but demonstrated varying expression in subsequent stages. Glutathione transferases (GSTs) are critical for protecting cells from oxidative damage when they are under stress. Salinity also had a big effect on secondary metabolism, which included DEGs that were involved in making flavonoids and other metabolites.

This research elucidates the molecular foundation of tolerance, potentially informing breeding initiatives focused on creating salt-resistant rice cultivars. The identified DEGs, especially those associated with critical pathways such as ion transport, transcriptional regulation, and secondary metabolism, present prospective targets for genetic enhancement. Future research seeks to delineate all the elements implicated in the perception and transduction of salt stress signals. The results help us understand how rice responds to salinity stress better and show how important it is to use integrative transcriptomic approaches to figure out how plants tolerate stress.

## 5. Conclusions

Salt stress has significant effects on both the Xiangxiuzhan and Changxiang varieties, which have different tolerances at different growth stages. The germination rates suggest that the CXG variety had significantly higher salt tolerance than the XXZ variety. However, the XXZ variety had a better chance of surviving than the CXG variety. Transcriptome analysis showed that salt stress caused a lot of changes in gene expression. CXG had more DEGs in the leaves and roots than in the grains, but XXZ had more DEGs in the grains. Functional annotation of DEGs through GO analysis indicated that stress-responsive genes were primarily enriched in binding and catalytic activities, with significant participation in biological and molecular processes. This finding will help future genetic studies on rice to enquire into the development of salt-tolerant rice varieties.

## Figures and Tables

**Table 1:** Sample information for the Xiangxiuzhan and Changxiang rice varieties under salt stress.

Cultivar	Tissue	Condition	Timepoint	NaCl Concentration	Replications	Mapping of Each Sample Code	
						Control Group	Treatment Group
Xiangxiuzhan (XXZ)	Grain	Growth	After 3 days of treatment	100 mM NaCl	3	CCK1, CCK2, CCK3	CNa1, CNa2, CNa3
	Leaf					CCKS1, CCKS2, CCKS3	CNaS1, CNaS2, CNaS3
	Root					CCKR1, CCKR2, CCKR3	CNaR1, CNaR2, CNaR3
Changxiang (CXG)	Grain					XCK1, XCK2, XCK3	XNa1, XNa2, XNa3
	Leaf					XCKS1, XCKS2, XCKS3	XNaS1, XNaS2, XNaS3
	Root					XCKR1, XCKR2, XCKR3	XNaR1, XNaR2, XNaR3

## Data Availability

The original contributions presented in this study are included in the article/Supplementary Materials. Further inquiries can be directed to the corresponding authors.

## Supplementary Materials

The following supporting information can be downloaded at https://www.mdpi.com/article/10.3390/cimb48030321/s1.

## References

[1] Zhu J. (2021). Plant salt tolerance. Trends Plant Sci..

[2] Tester M., Davenport R. (2003). Na^+^ Tolerance and Na+ Transport in Higher Plants. Ann. Bot..

[3] Tedeschi A., Xue X. (2025). Crop Response to Soil and Water Salinity. Soil Syst..

[4] Tang H., Du L., Xia C., Luo J. (2024). Bridging gaps and seeding futures: A synthesis of soil salinization and the role of plant-soil interactions under climate change. iScience.

[5] Munns R., Gilliham M. (2015). Salinity Tolerance of Crops—What Is the Cost?. New Phytol..

[6] Munns R., Tester M. (2008). Mechanisms of Salinity Tolerance. Annu. Rev. Plant Biol..

[7] Sade N., Del Mar Rubio-Wilhelmi M., Umnajkitikorn K., Blumwald E. (2018). Stress-Induced Senescence and Plant Tolerance to Abiotic Stress. J. Exp. Bot..

[8] Shrivastava P., Kumar R. (2015). Soil Salinity: A Serious Environmental Issue and Plant Growth Promoting Bacteria as One of the Tools for Its Alleviation. Saudi J. Biol. Sci..

[9] Mukhopadhyay R., Sarkar B., Jat H.S., Sharma P.C., Bolan N.S. (2021). Soil Salinity under Climate Change: Challenges for Sustainable Agriculture and Food Security. J. Environ. Manag..

[10] FAO (2021). FAO Global Map of Salt Affected Soils Version 1.0 (2021). https://www.fao.org/soils-portal/data-hub/soil-maps-and-databases/global-map-of-salt-affected-soils/en/.

[11] Munns R., Passioura J.B., Colmer T.D., Byrt C.S. (2020). Osmotic Adjustment and Energy Limitations to Plant Growth in Saline Soil. New Phytol..

[12] Hu Y., Schmidhalter U. (2004). Limitation of Salt Stress to Plant Growth. Plant Toxicology.

[13] Arif Y., Singh P., Siddiqui H., Bajguz A., Hayat S. (2020). Salinity Induced Physiological and Biochemical Changes in Plants: An Omic Approach towards Salt Stress Tolerance. Plant Physiol. Biochem..

[14] Hameed A., Ahmed M.Z., Hussain T., Aziz I., Ahmad N., Gul B., Nielsen B.L. (2021). Effects of Salinity Stress on Chloroplast Structure and Function. Cells.

[15] Ma Y., Dias M.C., Freitas H. (2020). Drought and Salinity Stress Responses and Microbe-Induced Tolerance in Plants. Front. Plant Sci..

[16] Shaughnessy C.A. (2015). Physiological Effects of Aquatic Hypercarbia on Seawater Acclimation in the White Sturgeon (*Acipenser Transmontanus*). Master’s Thesis.

[17] Wang Y., Li J. (2005). The Plant Architecture of Rice (*Oryza Sativa*). Plant Mol. Biol..

[18] Zeng L., Shannon M.C., Lesch S.M. (2001). Timing of Salinity Stress Affects Rice Growth and Yield Components. Agric. Water Manag..

[19] Sultana N., Ikeda T., Kashem M.A. (2001). Effect of Foliar Spray of Nutrient Solutions on Photosynthesis, Dry Matter Accumulation and Yield in Seawater-Stressed Rice. Environ. Exp. Bot..

[20] Pandit A., Rai V., Bal S., Sinha S., Kumar V., Chauhan M., Gautam R.K., Singh R., Sharma P.C., Singh A.K. (2010). Combining QTL Mapping and Transcriptome Profiling of Bulked RILs for Identification of Functional Polymorphism for Salt Tolerance Genes in Rice (*Oryza Sativa* L.). Mol. Genet. Genom..

[21] Geng L., Zhang W., Zou T., Du Q., Ma X., Cui D., Han B., Zhang Q., Han L. (2023). Integrating Linkage Mapping and Comparative Transcriptome Analysis for Discovering Candidate Genes Associated with Salt Tolerance in Rice. Front. Plant Sci..

[22] International Rice Genome Sequencing Project (2005). The map-based sequence of the rice genome. Nature.

[23] Wang J., Zhu J., Zhang Y., Fan F., Li W., Wang F., Zhong W., Wang C., Yang J. (2018). Comparative Transcriptome Analysis Reveals Molecular Response to Salinity Stress of Salt-Tolerant and Sensitive Genotypes of Indica Rice at Seedling Stage. Sci. Rep..

[24] De Abreu Neto J.B., Frei M. (2016). Microarray Meta-Analysis Focused on the Response of Genes Involved in Redox Homeostasis to Diverse Abiotic Stresses in Rice. Front. Plant Sci..

[25] Sengupta S., Majumder A.L. (2009). Insight into the Salt Tolerance Factors of a Wild Halophytic Rice, Porteresia Coarctata: A Physiological and Proteomic Approach. Planta.

[26] Khan M.H.U., Malook I., Atlas A., Jan M., Ullah S., Shah G. (2014). The Effect of Sodium Chloride (NaCl) Stress on Seed Germination and Seedling Growth of Rice (*Oryza Sativa* L.). J. Bio-Molecular Sci..

[27] Sun Y., Li J., Xing J., Yu X., Lu Y., Xu W., Zhao N., Liu Z., Guo Z. (2022). Evaluation of Salt Tolerance in Common Vetch (*Vicia Sativa* L.) Germplasms and the Physiological Responses to Salt Stress. J. Plant Physiol..

[28] Chen C., Chen H., Zhang Y., Thomas H.R., Frank M.H., He Y., Xia R. (2020). TBtools: An Integrative Toolkit Developed for Interactive Analyses of Big Biological Data. Mol. Plant.

[29] Roy S.J., Negrão S., Tester M. (2014). Salt Resistant Crop Plants. Curr. Opin. Biotechnol..

[30] Cotsaftis O., Plett D., Shirley N., Tester M., Hrmova M. (2012). A Two-Staged Model of Na^+^ Exclusion in Rice Explained by 3d Modeling of HKT Transporters and Alternative Splicing. PLoS ONE.

[31] Formentin E., Sudiro C., Perin G., Riccadonna S., Barizza E., Baldoni E., Lavezzo E., Stevanato P., Sacchi G.A., Fontana P. (2018). Transcriptome and Cell Physiological Analyses in Different Rice Cultivars Provide New Insights into Adaptive and Salinity Stress Responses. Front. Plant Sci..

[32] Ismail A.M., Horie T. (2017). Genomics, Physiology, and Molecular Breeding Approaches for Improving Salt Tolerance. Annu. Rev. Plant Biol..

[33] Kobayashi N.I., Yamaji N., Yamamoto H., Okubo K., Ueno H., Costa A., Tanoi K., Matsumura H., Fujii-Kashino M., Horiuchi T. (2017). OsHKT1;5 Mediates Na^+^ Exclusion in the Vasculature to Protect Leaf Blades and Reproductive Tissues from Salt Toxicity in Rice. Plant J..

[34] Liu Y., Liu B., Luo K., Yu B., Li X., Zeng J., Chen J., Xia R., Xu J., Liu Y. (2024). Genomic Identification and Expression Analysis of Acid Invertase (AINV) Gene Family in Dendrobium Officinale Kimura et Migo. BMC Plant Biol..

[35] Peng X., Liu H., Chen P., Tang F., Hu Y., Wang F., Pi Z., Zhao M., Chen N., Chen H. (2019). A Chromosome-Scale Genome Assembly of Paper Mulberry (*Broussonetia Papyrifera*) Provides New Insights into Its Forage and Papermaking Usage. Mol. Plant.

[36] Song C., Zhang Y., Zhang W., Manzoor M.A., Deng H., Han B. (2023). The Potential Roles of Acid Invertase Family in Dendrobium Huoshanense: Identification, Evolution, and Expression Analyses under Abiotic Stress. Int. J. Biol. Macromol..

[37] Muthuramalingam P., Jeyasri R., Rakkammal K., Satish L., Shamili S., Karthikeyan A., Valliammai A., Priya A., Selvaraj A., Gowri P. (2022). Multi-Omics and Integrative Approach towards Understanding Salinity Tolerance in Rice: A Review. Biology.

[38] Jha U.C., Bohra A., Jha R., Parida S.K. (2019). Salinity Stress Response and ‘Omics’ Approaches for Improving Salinity Stress Tolerance in Major Grain Legumes. Plant Cell Rep..

