# Supplementary material for: Comparative Transcriptomic Analysis Reveals Salt Stress Adaptation Mechanisms in Cultivated Rice Varieties (Oryza sativa)
Source: Curr Issues Mol Biol. 2026 Mar 18;48(3):321. doi: 10.3390/cimb48030321 (PMC13025425; doi:10.3390/cimb48030321)
Supplement: Supplementary file 1 [file cimb-48-00321-s001.zip › cimb-4149817-supplementary.pdf]

Supplementary

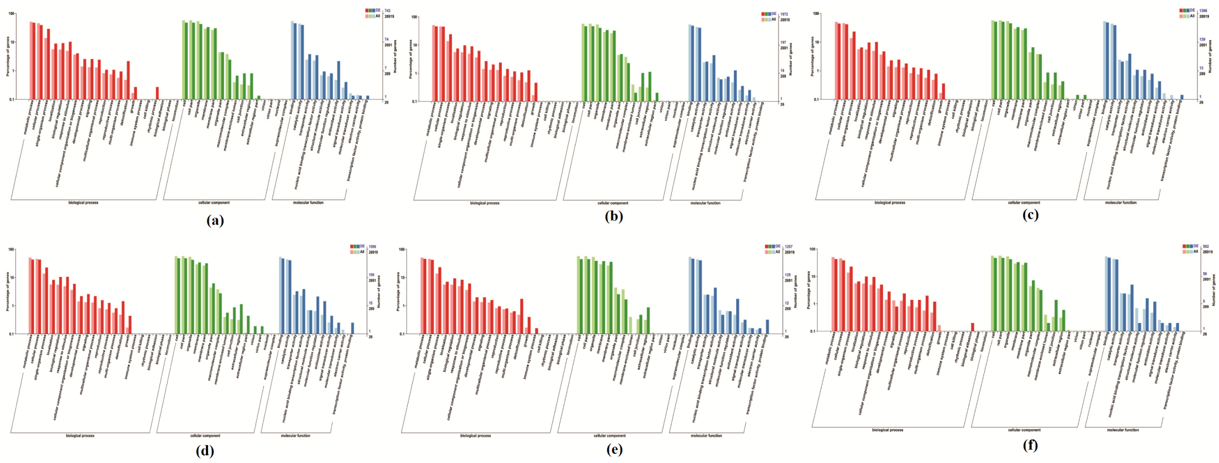

Supplementary Figure S1: GO classification map of salt stress response genes in seeds, leaves, and roots of CXG and XXZ variety. Here, (a) indicates seeds of CXG groups, (b) indicates leaves CXG group, (c) indicates roots of CXG groups, (d) indicates seeds of XXZ groups, (e) indicates leaves XXZ group, (f) indicates roots of XXZ groups.
